# Autophagy regulates rRNA synthesis

**DOI:** 10.1080/19491034.2022.2114661

**Published:** 2022-08-21

**Authors:** Yinfeng Xu, Wei Wan

**Affiliations:** a Laboratory of Basic Biology, Hunan First Normal University, Changsha, Hunan, China; b Department of Biochemistry, and Department of Thoracic Surgery of Sir Run Run Shaw Hospital, Zhejiang University School of Medicine, Hangzhou, Zhejiang, China

**Keywords:** Autophagy, MTORC1, rDNA, rRNA, SQSTM1/p62

## Abstract

Autophagy has emerged as a key regulator of cell metabolism. Recently, we have demonstrated that autophagy is involved in RNA metabolism by regulating ribosomal RNA (rRNA) synthesis. We found that autophagy-deficient cells display much higher 47S precursor rRNA level, which is caused by the accumulation of SQSTM1/p62 (sequestosome 1) but not other autophagy receptors. Mechanistically, SQSTM1 accumulation potentiates the activation of MTOR (mechanistic target of rapamycin kinase) complex 1 (MTORC1) signaling, which facilitates the assembly of RNA polymerase I pre-initiation complex at ribosomal DNA (rDNA) promoter regions and leads to the activation of rDNA transcription. Finally, we showed that SQSTM1 accumulation is responsible for the increase in protein synthesis, cell growth and cell proliferation in autophagy-deficient cells. Taken together, our findings reveal a regulatory role of autophagy and autophagy receptor SQSTM1 in rRNA synthesis and may provide novel mechanisms for the hyperactivated rDNA transcription in autophagy-related human diseases.

**Abbreviations:** 5-FUrd: 5-fluorouridine; LAP: MAP1LC3/LC3-associated phagocytosis; MAP1LC3/LC3: microtubule associated protein 1 light chain 3; MTOR: mechanistic target of rapamycin kinase; PIC: pre-initiation complex; POLR1: RNA polymerase I; POLR1A: RNA polymerase I subunit A; rDNA: ribosomal DNA; RRN3: RRN3 homolog, RNA polymerase I transcription factor; rRNA: ribosomal RNA; SQSTM1/p62: sequestosome 1; TP53INP2: tumor protein p53 inducible nuclear protein 2; UBTF: upstream binding transcription factor.

## Introduction

Macroautophagy (hereafter called autophagy) is a highly conserved degradation pathway in eukaryotic cells, which helps maintain cellular homeostasis by sequestering the unwanted intracellular materials or the invading pathogens into double-membrane autophagosomes and delivering them to the lysosomes for digestion [1–3]. Accumulating evidence has demonstrated that autophagy is intensively involved in the regulation of cell metabolism by controlling the breakdown of macromolecules, such as proteins and lipids, and the turnover of organelles, such as mitochondria, ribosomes and peroxisomes [4,5]. However, whether autophagy plays a role in RNA metabolism in the cell remains largely unknown. In a recent study, we found that autophagy deficiency upregulates cellular ribosomal RNA (rRNA) level by activating ribosomal DNA (rDNA) transcription, which then facilitates protein synthesis, cell growth and cell proliferation [6].

## Autophagy controls 47S rRNA level

All kinds of rRNAs, such as 5.8S rRNA, 18S rRNA and 28S rRNA, derived from the precursor rRNAs, account for the most cellular nascent RNAs [7–9]. All the rRNAs are indispensable components for the assembly of ribosomes, which are the only factories for protein synthesis in the cell [7–9].

To explore whether autophagy is involved in RNA metabolism, we began by examining the level of 47S rRNA, the precursor rRNA of 5.8S rRNA, 18S rRNA and 28S rRNA in the cell. Interestingly, 47S rRNA level is upregulated in cells with deletion of *ATG*5 or *ATG*7 [6], which are essential for MAP1LC3/LC3 (microtubule associated protein 1 light chain 3) lipidation and autophagosome formation [10]. Considering that LC3 lipidation also participates in autophagy-independent events, such as LC3-associated phagocytosis (LAP), we examined 47S rRNA level in cells with deletion of *RUBCN* (rubicon autophagy regulator), required for LAP but not autophagy, or *ATG16L1*, another indispensable component for LC3 lipidation [10,11]. Obviously, knockout of *ATG16L1* but not *RUBCN* increases cellular 47S rRNA level [6]. In addition, we found that 47S rRNA level is also upregulated in cells with deletion of *ULK1*, which is required for ATG5- and ATG7-dependent and independent autophagy [6].

Finally, to test whether 47S rRNA undergoes autophagic degradation, we measured its decay rate in *ATG16L1*-deficient cells and found that the half-time remains unchanged [6], suggesting that 47S rRNA may not be the direct substrate of autophagy.

## Autophagy receptor SQSTM1 activates rDNA transcription

The recruitment of different autophagic substrates, such as protein aggregates and damaged organelles, to the autophagosomes relies on various autophagy receptors, which link the specific autophagic substrates to the autophagy machinery and undergo degradation with the substrates in the lysosomes [12,13].

Given that autophagy receptors are markedly accumulated in autophagy-deficient cells, we then tested whether some autophagy receptors are involved in the regulation of 47S rRNA level in the cell. Knockout of the five major autophagy receptors, namely *SQSTM1, NBR1, TAX1BP1, OPTN* and *CALCOCO2/NDP52* significantly decreased cellular 47S rRNA level [6]. However, re-introduction of SQSTM1 but not other autophagy receptors rescued the decrease of 47S rRNA level in the cells [6]. In addition, knockout of *SQSTM1* also abolished the increase of 47S rRNA level in cells with *ATG7* deletion [6]. Furthermore, we found that binding to LC3 or ubiquitinated substrates is not required for SQSTM1 to elevate cellular 47S rRNA level [6], suggesting an autophagy receptor-independent function of SQSTM1 in this process.

As 47S rRNA seems not to be the direct substrate of autophagy, we then investigated whether autophagy regulates the synthesis of 47S rRNA. We performed an *in situ* run-on assay to check the incorporation of 5-fluorouridine (5-FUrd) into nascent RNAs, which indicates local RNA synthesis activity directly [14]. Notably, SQSTM1 overexpression promoted 5-FUrd incorporation at the nucleolus [6], where rRNA synthesis takes place. These data suggest that accumulated SQSTM1 may upregulate cellular 47S rRNA level by activating rRNA synthesis in autophagy-deficient cells.

47S rRNA is transcribed from rDNA by RNA polymerase I (POLR1) at the nucleolus [7,15–17]. Activation of rDNA transcription requires the assembly of POLR1 pre-initiation complex (PIC) at rDNA promoters [7,15–17]. Besides POLR1, several proteins, such as UBTF (upstream binding transcription factor), RRN3 (RRN3 homolog, RNA polymerase I transcription factor) and TP53INP2 (tumor protein p53 inducible nuclear protein 2), are also involved in the assembly of POLR1 PIC [7,15–18].

We examined the promoter activity of rDNA and found that knockout of *ATG16L1* but not *RUBCN* increases rDNA promoter activity [6], suggesting that rDNA transcription is activated in autophagy-deficient cells. Interestingly, further knockout of *SQSTM1* was sufficient to abolish the increase of rDNA promoter activity in *ATG7*-deficient cells [6]. In addition, we found that only SQSTM1, but not other autophagy receptors, is sufficient to increase rDNA promoter activity [6]. We then checked the rDNA promoter binding of UBTF and POLR1A and found that SQSTM1 is also sufficient and necessary to enhance the binding of UBTF and POLR1A to rDNA promoter regions in autophagy-deficient cells [6]. Furthermore, we examined the assembly of POLR1A PIC by checking the interaction between UBTF and POLR1A, and found that accumulated SQSTM1 is responsible for the increased UBTF-POLR1A interaction in autophagy-deficient cells [6].

These findings together suggest that accumulated SQSTM1 mediates rDNA transcription activation in autophagy-deficient cells.

## Autophagy regulates MTORC1 signaling through a SQSTM1-dependent manner

Besides autophagy, SQSTM1 is also involved in the regulation of many cellular signaling pathways, including MTORC1 pathway [19–22]. In response to various intracellular stimuli or environmental cues, such as nutrients or growth factors, MTORC1 is recruited to the lysosomal surface for activation [23,24]. MTORC1 has been demonstrated to regulate a variety of metabolic processes, such as rDNA transcription [16,18,23,24]. Interestingly, SQSTM1 is reported to interact with MTORC1 to potentiate its activation by promoting its recruitment to the lysosomal surface [19]. Notably, the role of SQSTM1 in the regulation of MTORC1 signaling is independent of its function as an autophagy receptor [19].

To investigate the mechanism underlying autophagy deficiency-induced rDNA transcription activation, we checked whether autophagy deficiency regulates MTORC1 activity. Obviously, cells with deletion of *ATG5* or *WIPI2* displayed much higher MTORC1 activity [6]. Knockout of *SQSTM1* reversed the increased MTORC1 activity in the cells with *ATG7* deletion [6]. In addition, overexpression of SQSTM1, but not other autophagy receptors, was sufficient to increase MTORC1 activity [6]. However, in *Tsc2*-deficient cells, in which MTORC1 is constitutively activated, SQSTM1 overexpression failed to further increase MTORC1 activity [6]. Consistent with this, SQSTM1 also failed to further elevate 47S rRNA level in these cells [6]. Moreover, inactivation of MTORC1 signaling by serum starvation or rapamycin treatment completely abolished the enhanced interaction between UBTF and POLR1A induced by SQSTM1 overexpression [6].

Taken together, these data suggest that SQSTM1-mediated increase of MTORC1 activity is responsible for the assembly of POLR1A PIC and the activation of rDNA transcription in autophagy-deficient cells.

## Autophagy regulates protein synthesis, cell growth and cell proliferation

rDNA transcription is the rate-limiting step for ribosome biogenesis, which is the prerequisite for a number of cellular processes, including protein synthesis, cell growth and cell proliferation [16,17].

To investigate the biological significance of autophagy deficiency-induced activation of rDNA transcription, we firstly checked the protein synthesis rate in autophagy-deficient cells. By detecting the amount of nascent proteins, we found that the protein synthesis rate is increased in *ATG7*-deficient cells and *SQSTM1* knockout completely reverses the increase [6]. In addition, we examined cell growth and cell proliferation by measuring cell diameter and counting cell number, respectively. The results showed that SQSTM1 overexpression is sufficient to promote cell growth and cell proliferation, and the promoting effect is abolished by rapamycin treatment [6].

Therefore, these results suggest that accumulated SQSTM1 is required for autophagy deficiency to promote protein synthesis, cell growth and cell proliferation.

## Concluding remarks

**Figure 1.**
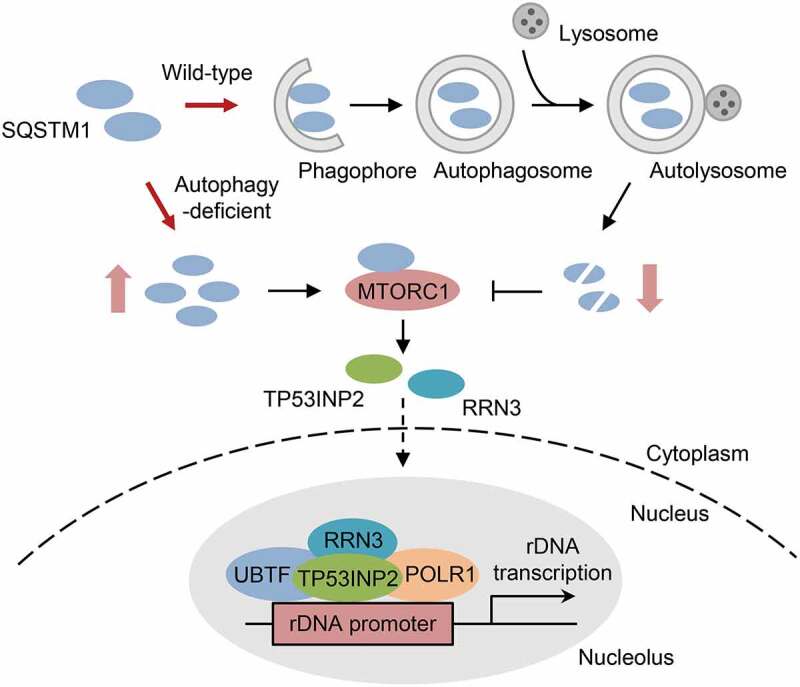
A summary of the role of autophagy in rDNA transcription. In wild-type cells, autophagy receptor SQSTM1 binds to autophagic substrates and targets them to phagophore, which expands and forms double-membrane autophagosome. The sealed autophagosomes then fuse with the lysosomes and cause the degradation of the engulfed contents, which decreases cellular SQSTM1 level and MTORC1 activity, leading to the cytoplasmic retention of TP53INP2 and RRN3 and the inhibition of rDNA transcription. Conversely, autophagy deficiency causes SQSTM1 accumulation and potentiates MTORC1 activation in the cell, which promotes the nuclear and nucleolar translocation of TP53INP2 and RRN3 from the cytoplasm. Then, nucleolar TP53INP2 and RRN3 assemble into POLR1A PIC with other components at rDNA promoter regions, leading to the activation of rDNA transcription.

Based on the findings from our group and previous studies, herein we propose a working model for autophagy deficiency-induced rDNA transcription activation (Figure 1). As an autophagy receptor, SQSTM1 is sequestered into autophagosomes along with the substrates for subsequent lysosome-dependent degradation in wild-type cells, which keeps the low protein level of SQSTM1 and decreases the activity of MTORC1, leading to the strong cytoplasmic distribution of TP53INP2 and RRN3 and the suppression of rDNA transcription (Figure 1). However, in autophagy-deficient cells, accumulated SQSTM1 potentiates the activation of MTORC1 signaling, which stimulates the nuclear and nucleolar translocation of TP53INP2 and RRN3 from the cytoplasm and promotes the formation of POLR1A PIC at rDNA promoter regions, leading to rDNA transcription activation (Figure 1).

By elucidating the function and mechanism of autophagy in rDNA transcription, we uncover a regulatory role of autophagy and an autophagy receptor-independent function of SQSTM1 in RNA metabolism. Given that both autophagy dysregulation and hyperactivated rDNA transcription are involved in the pathogenesis of various human diseases, including many types of cancers [7,25–27], our findings may provide novel mechanisms and potential therapeutic targets for these autophagy-related diseases.
